# Correlated Time-0 and Hot-Carrier Stress Induced FinFET Parameter Variabilities: Modeling Approach

**DOI:** 10.3390/mi11070657

**Published:** 2020-06-30

**Authors:** Alexander Makarov, Philippe Roussel, Erik Bury, Michiel Vandemaele, Alessio Spessot, Dimitri Linten, Ben Kaczer, Stanislav Tyaginov

**Affiliations:** 1 Institute for Microelectronics, TU Wien, 1040 Vienna, Austria; 2 Imec, 3001 Leuven, Belgium; Philippe.Roussel@imec.be (P.R.); Erik.Bury@imec.be (E.B.); Michiel.Vandemaele@imec.be (M.V.); Alessio.Spessot@imec.be (A.S.); Dimitri.Linten@imec.be (D.L.); Ben.Kaczer@imec.be (B.K.); Stanislav.Tyaginov@imec.be (S.T.); 3 Department of Electrical Engineering (ESAT), KU Leuven, 3001 Leuven, Belgium; 4 Ioffe Institute, 194021 St. Petersburg, Russia

**Keywords:** hot-carrier degradation, random dopants, variability, physical modeling, FinFETs, carrier transport, interface traps, random traps

## Abstract

We identify correlation between the drain currents in pristine n-channel FinFET transistors and changes in time-0 currents induced by hot-carrier stress. To achieve this goal, we employ our statistical simulation model for hot-carrier degradation (HCD), which considers the effect of random dopants (RDs) on HCD. For this analysis we generate a set of 200 device instantiations where each of them has its own unique configuration of RDs. For all “samples” in this ensemble we calculate time-0 currents (i.e., currents in undamaged FinFETs) and then degradation characteristics such as changes in the linear drain current and device lifetimes. The robust correlation analysis allows us to identify correlation between transistor lifetimes and drain currents in unstressed devices, which implies that FinFETs with initially higher currents degrade faster, i.e., have more prominent linear drain current changes and shorter lifetimes. Another important result is that although at stress conditions the distribution of drain currents becomes wider with stress time, in the operating regime drain current variability diminishes. Finally, we show that if random traps are also taken into account, all the obtained trends remain the same.

## 1. Introduction

One of the main problems plaguing performance of modern ultra-scaled field-effect transistors (FETs) is variability, which has multiple sources: random dopants (RDs) [1], perturbation in material properties [2,3], oxide thickness fluctuations [4,5], etc. This variability manifests itself already in pristine devices and therefore is named “time-0 variability”. In a very similar manner, build-up of defects during stress is a stochastic process and therefore degradation in ultra-scaled devices should be described statistically as time-dependent variability [6].

A link between these two types of variabilities has been a subject of previous studies. Investigations conducted by Kerber et al. [7,8] resulted in a correlation between distributions of the threshold voltage $V_{\mathrm{th}}$ in unstressed devices and threshold voltage changes $\Delta V_{\mathrm{th}}$ induced by bias temperature instability (BTI). Quite to the contrary, Angot et al. [9] and Hussin et al. [10] did not reveal any correlation between time-0 $V_{\mathrm{th}}$ values and their BTI-induced shifts $\Delta V_{\mathrm{th}}$. The latter group has been very actively studying the correlation between time-0 and stress induced variabilities also in the context of self-heating [11] and random dopant induced percolation paths in FETs [12].

As for hot-carrier degradation (HCD), which has been repeatedly declared to be the most detrimental reliability concern in ultra-scaled FETs [13,14], although HC induced variability was a subject of experimental [15,16,17,18,19,20,21] and modeling [22,23,24,25,26,27,28] studies, to the best of our knowledge there is a limited number of publications devoted to correlation between time-0 and HC stress induced transistor parameter distributions [29,30,31], and no simulation studies of this correlation have been performed so far. Schlünder et al. [29] reported a strong correlation between parameters in the $\{V_{\mathrm{th}}\left(t=0\right)$, $\Delta V_{\mathrm{th}}\}$ and $\{I_{d}\left(t=0\right)$, $\Delta I_{d}\}$ tuples (here *t* is the stress time, while $I_{d}$ is the drain current). This study showed that devices with initially higher drain currents degrade faster and therefore have larger $\Delta I_{d}$ values and shorter device lifetimes τ. A very similar trend was reported also by Ramey and co-authors [30], i.e., devices with initially higher $V_{\mathrm{th}}$ values demonstrated slightly larger $\Delta V_{\mathrm{th}}$ changes. Finally, the work of Federspiel et al. [31] also reports correlation between time-0 threshold voltages and their HC induced drifts; the authors also identified correlation between changes of the threshold voltage and the drain current during HCD and this finding is consistent with the results from [30].

However, a physics-based modeling framework for HCD which can capture these experimental trends is still missing. In this work, we perform the first theoretical study of the correlation between time-0 values of the linear and saturation drain currents ($I_{d,\mathrm{lin}}^{\left(0\right)}$ and $I_{d,\mathrm{sat}}^{\left(0\right)}$, respectively) in n-channel FinFETs and relative drain current changes ($\Delta I_{d,\mathrm{lin}}$ and $\Delta I_{d,\mathrm{sat}}$) induced by HC stress. For this we employ our approach to stochastic HCD modeling [23,25,26]. We consider the roles of impacts of random dopants and random traps on this correlation.

## 2. The Modeling Framework

**Figure 1:**
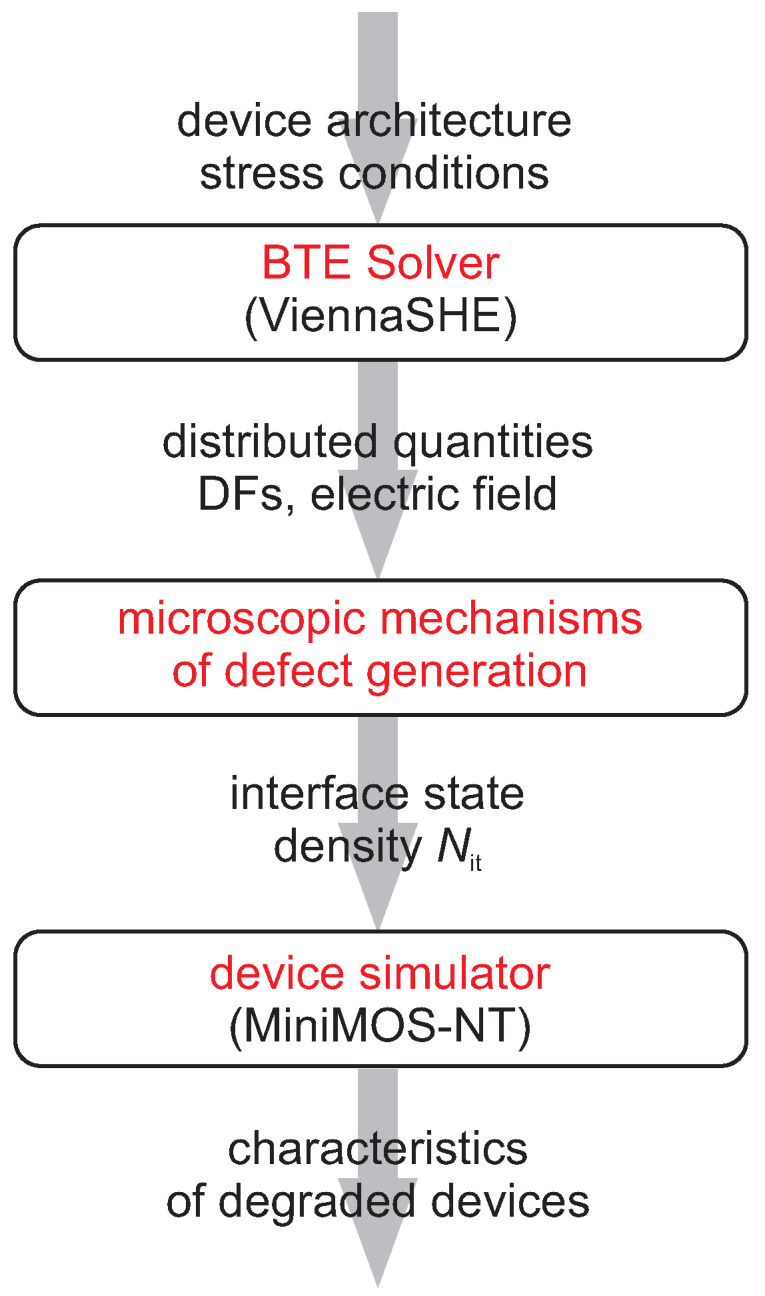
The schematic representation of our modeling framework which covers and links three main sub-tasks of the problem of hot-carrier degradation: modeling of carrier transport, modeling of the defect generation rates, and simulations of the degraded devices.

The stochastic version of our HCD model (which is described in greater detail in [23,25]) is derived from the deterministic approach to HCD simulations validated against data acquired in devices of different types, which include planar FETs [32,33], power laterally-diffused metal-oxide semiconductor transistors [34,35], FinFETs [36] and nanowire FETs [37]. To accurately describe the complex physical picture behind HCD, this approach captures and links carrier transport, a microscopic description of defect generation rates, and modeling of the degraded devices, see Figure 1, which sketches the model structure.

### 2.1. Carrier Transport Modeling

For carrier transport modeling we use the deterministic solver of the Boltzmann transport equation (BTE) ViennaSHE [38,39,40,41], which is based on the spherical harmonics expansion of the carrier energy distribution function (DF). ViennaSHE incorporates the real band structure of Si and models the rates of different scattering mechanisms including impact ionization, surface scattering, ionized impurity scattering as well as electron-phonon and carrier-carrier interactions. The effect of traps generated by HCD was recently incorporated into ViennaSHE [42]. ViennaSHE solves the bipolar BTE for a particular device architecture and specified stress or operating conditions. Note that for obtaining the carrier DFs one can use another transport simulator, as for example a stochastic BTE solver based on the Monte Carlo method (in the first version of our HCD model [43,44] we used the simulator MONJU based on the Mote Carlo approach to the BTE solution [45]).

### 2.2. Defect Generation

**Figure 2:**
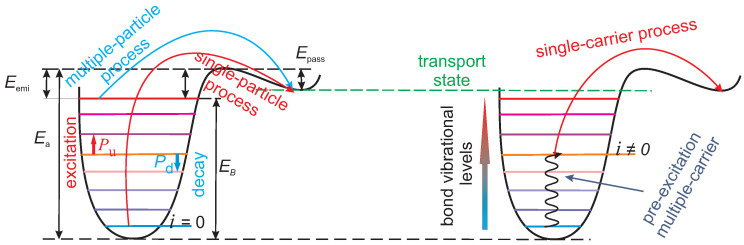
Single- and multiple-carrier mechanisms of Si-H bond rupture (left panel) and their superposition (right panel).

The carrier energy DFs are then used to calculate the rates of single- and multiple-carrier mechanisms (SC- and MC-mechanisms) of Si-H bond breakage (Figure 2). Under stress conditions with high drain voltages carriers can be accelerated up to energies exceeding the bonding energy of the Si-H bond which is $E_{a}\;=\;2.6-2.9$ eV [46,47,48] and therefore they are able to trigger bond rupture in a single collision. This scenario is called “single-carrier mechanism” of bond dissociation and was dominant in high-voltage devices and/or old technology nodes [49,50,51]. Under lower drain voltages these highly energetical carriers have low concentrations and therefore the single-carrier mechanism has a negligibly low rate. However, several cold carriers can subsequently bombard the bond and induce its multiple vibrational excitation, which eventually results in a bond rupture event; this scenario is named “multiple-carrier mechanism” and has high rates in ultra-scaled devices [52,53,54,55]. One can say that the single-carrier mechanism is driven by hot carriers, while the multiple-carrier process is associated with cold carriers. This is the main reason why we need to know how carriers are distributed over energy and solve the BTE to obtain the carrier DFs. If a bond is pre-heated by several cold carriers to some excited by still bonded state (Figure 2, right panel) with an index *i* and the energetical position $E_{i}$ the bond-breakage energy is reduced by the value $E_{i}$ and is effectively equal to $E_{a}-E_{i}$. Therefore, the probability of a single-carrier bond breakage event from this level *i* dramatically increases. Recently it was reported that in modern ultra-scaled devices the most probable path of bond dissociation is via a mixture of multiple- and single-carrier mechanisms of bond dissociation [33,56,57].

We model the Si-H bond within the truncated harmonic oscillator model, i.e., the bonding potential is parabolic and the eigenstates of the bond are equidistantly spaced with the distance between them being ℏω, see Figure 2. The dissociation reaction occurs via the stretching mode of the bond (another vibrational mode of the Si-H bond is the bending mode). This is because the bonding energy of this mode $E_{a}$ = 2.56 eV (Figure 2) corresponds to the bond-breakage energy measured during experiments of bond dissociation [48]; the corresponding parameter ℏω is equal to 0.25 eV. To model the rates of the SC- and MC-mechanisms we employ the formalism described in sufficient detail in [32,33,57,58]. The rates of both SC- and MC-mechanisms are determined by the carrier acceleration integral:

(1) $$\begin{array}{c} I_{\mathrm{SC}|\mathrm{MC}}\;=\;\int_{E_{\mathrm{th}}}^{\infty }f\left(\varepsilon \right)g\left(\varepsilon \right)\sigma_{\mathrm{SC}|\mathrm{MC}}\left(\varepsilon \right)v\left(\varepsilon \right)d\varepsilon . \end{array}$$

Here f(ε) is the carrier DF obtained with ViennaSHE, g(ε) the density-of-states of the corresponding carrier band, v(ε) the carrier group velocity and integration is performed over carrier energy ε. The Keldysh-like reaction cross section $\sigma_{\mathrm{SC}|\mathrm{MC}}\left(\varepsilon \right)$ for both processes is given by

(2) $$\begin{array}{c} \sigma_{\mathrm{SC}|\mathrm{MC}}\left(\varepsilon \right)\;=\;\sigma_{0,\mathrm{SC}|\mathrm{MC}}\;{\left[(\varepsilon -\varepsilon_{\mathrm{th}})/1\;\mathrm{eV}\right]}^{p_{\mathrm{SC}|\mathrm{MC}}}, \end{array}$$

with the parameters $p_{\mathrm{SC}}$ and $p_{\mathrm{MC}}$ being equal to 11 and 1 for SC- and MC-processes, correspondingly [43,58]. For the MC-mechanism the threshold energy is equal to the distance between the oscillator levels ℏω, while for the SC-mechanism occurring form the ground state of the bond (Figure 2, left panel) we assume $\varepsilon_{\mathrm{th}}\;=\;E_{a}$. The prefactors $\sigma_{0,\mathrm{SC}|\mathrm{MC}}$ are adjustable parameters of the model.

If the bond is preheated by several cold carriers (Figure 2, right panel) to a bonded state *i* the SC-mechanism rate reads

(3) $$I_{\mathrm{SC},i}\left(E_{a}\right)\;=\;\int f\left(\varepsilon \right)g\left(\varepsilon \right)\sigma_{0}{\left(\varepsilon -E_{a}+E_{i}\right)}^{p_{\mathrm{it},\mathrm{SP}}}v\left(\varepsilon \right)d\varepsilon$$

and the total bond rupture rate from this level is

(4) $$R_{\mathrm{SC},i}\;=\;w_{\mathrm{th}}\exp\left[-\left(E_{a}-E_{i}\right))/k_{B}T_{L}\right]+I_{\mathrm{SC},i},$$

where the first term is the rate of the thermal activation of the bond over the potential barrier between the level *i* and the transport mode (with the corresponding attempt rate $w_{\mathrm{th}}$) and $I_{\mathrm{SC},i}$ is the SC-process rate.

To model the bond dissociation kinetic we employ the system of rate equations for each eigenstate (the last bonded state is labeled as $N_{l}$, see Figure 2):

(5) $$\begin{array}{c} \frac{dn_{0}}{dt}\;=\;P_{d}n_{1}-P_{u}n_{0}-R_{a,0}n_{0}+{\tilde{R}}_{p,0}N_{\mathrm{it}}^{2}\;\\ \frac{dn_{i}}{dt}\;=\;P_{d}\left(n_{i+1}-n_{i}\right)-P_{u}\left(n_{i}-n_{i-1}\right)-R_{a,i}n_{i}+{\tilde{R}}_{p,i}N_{\mathrm{it}}^{2}\;\\ \frac{dn_{N_{l}}}{dt}\;=\;P_{u}n_{N_{l-1}}-P_{d}n_{N_{l}}-R_{a,N_{l}}n_{N_{l}}+{\tilde{R}}_{p,N_{l}}N_{\mathrm{it}}^{2}. \end{array}$$

In these equations, $n_{i}$ is the level occupation number and the rates $P_{u}$, $P_{d}$ correspond to bond excitation and deexcitation, $N_{\mathrm{it}}$ is the concentration of interface traps and ${\tilde{R}}_{p,i}$ is the normalized passivation rate.

The rates of bond heating and cooling are

(6) $$\begin{array}{c} P_{u}\;=\;\omega_{e}\exp\left(-\hbar \omega /k_{B}T_{L}\right)+I_{\mathrm{MC}},\;\\ P_{d}\;=\;\omega_{e}+I_{\mathrm{MC}}, \end{array}$$

with $\omega_{e}$ being the vibrational frequency of the bond and which is equal to the reciprocal vibrational lifetime $\tau_{e}$ (for the stretching mode this lifetime is equal to 1.5 ns at room temperature [59]) and the term $I_{\mathrm{MC}}$ is the rate of the MC-process calculated by (1).

We solve the system (5) assuming the hierarchy of time scales, i.e., taking into account that bond heating and cooling processes have much shorter characteristic times than the bond rupture and passivation mechanisms. Within this approach the system reduces to a single differential equation:

(7) $$\frac{dN_{\mathrm{it}}}{dt}\;=\;\left(N_{0}-N_{\mathrm{it}}\right)R_{a}-N_{it}^{2}{\tilde{R}}_{p},$$

where $N_{0}$ is the concentration of electrically inactive Si-H bonds at the interface and $R_{a}$ is the cumulative bond breakage rate:

(8) $$\begin{array}{c} R_{a}\;=\;\frac{1}{k}\sum_{i}R_{a,i}{\left(\frac{P_{u}}{P_{d}}\right)}^{i}, \end{array}$$

where summation is performed over all bonded levels and bond rupture rates are weighted with the corresponding occupation numbers

(9) $$n_{i}\;=\;\frac{1}{k}{\left(\frac{P_{u}}{P_{d}}\right)}^{i}.$$

Here *k* is the prefactor which ensures normalization $\sum n_{i}\;=\;1$ and is equal:

(10) $$k\;=\;\sum_{i}{\left(\frac{P_{u}}{P_{d}}\right)}^{i}.$$

The passivation rate, which is determined as ${\tilde{R}}_{p}\;=\;\sum_{i}{\tilde{R}}_{p,i}$, can be simplified and modeled as a thermal activation over a single barrier $E_{\mathrm{pass}}$:

(11) $$R_{p}\;=\;\nu_{p}\exp\left(-E_{\mathrm{pass}}/k_{B}T_{L}\right),$$

where $\nu_{p}$ is the attempt rate. As for the normalized rate entering (5) it is determined as ${\tilde{R}}_{p}\;=\;R_{p}/N_{0}$. Following experimental papers [60,61,62] we use $E_{\mathrm{pass}}\;=\;1.5$ eV.

The system of rate equations (5) simplified as (7) has an analytical solution:

(12) $$\begin{array}{c} N_{it}\left(t\right)\;=\;\frac{\sqrt{R_{a}^{2}/4+N_{0}R_{a}{\tilde{R}}_{p}}}{{\tilde{R}}_{p}}\;\frac{1-\tilde{f}\left(t\right)}{1+\tilde{f}\left(t\right)}-\frac{R_{a}}{2{\tilde{R}}_{p}},\\ \tilde{f}\left(t\right)\;=\;\frac{\sqrt{R_{a}^{2}/4+N_{0}R_{a}{\tilde{R}}_{p}}-R_{a}/2}{\sqrt{R_{a}^{2}/4+N_{0}R_{a}{\tilde{R}}_{p}}+R_{a}/2}\;\;\exp\left(-2t\sqrt{R_{a}^{2}/4+N_{0}R_{a}{\tilde{R}}_{p}}\right). \end{array}$$

Here the time dependencies of $N_{\mathrm{it}}$ is determined by the component $\tilde{f}\left(t\right)$.

Due to the amorphous nature of SiO_2_ the bonding energy $E_{a}$ is a fluctuating quantity and in the model it is assumed to follow the normal distribution with the mean value $\langle E_{a}\rangle$ = 2.56 eV [46] and a certain standard deviation $\sigma_{E}$ which depends on the particular technology node (an adjustable parameter of the model).

### 2.3. Modeling of the Degraded Devices

The interface traps generated during HC stress are amphoteric and can capture electrons and holes. Since the concentration $N_{\mathrm{it}}$ varies with the coordinate along the $\mathrm{Si}/\mathrm{Si}O_{2}$ interface the corresponding charge incorporated into the transistor is also non-uniformly distributed. The impact of this charge on device performance is twofold: (1) charged traps locally perturb the device potential profile and (2) scatter carriers, thereby degrading the mobility.

To model these two aspects we use the device and circuit simulator MiniMOS-NT [63] which employs drift-diffusion and energy transport schemes as simplified approaches to the BTE solution. MiniMOS-NT also has a solver for the Poisson equation which captures the effect of interface traps on the device electrostatics. As for the reduction in the carrier mobility we use an empirical model [64,65]:

(13) $$\mu_{\mathrm{it}}\;=\;\frac{\mu_{0}}{1+\alpha_{\mathrm{it}}N_{\mathrm{it}}\exp\left(-r/r_{\mathrm{ref}}\right)},$$

where $\mu_{0}$ corresponds to the mobility of the pristine device, $\alpha_{\mathrm{it}}$ is the parameter which determines the magnitude of the impact of interface traps on the carrier mobility, *r* the distance from the carrier to the $\mathrm{Si}/\mathrm{Si}O_{2}$ interface, and $r_{\mathrm{ref}}$ the characteristic length at which the carriers “feel” the interface charges. Strictly speaking, the quantities $\alpha_{\mathrm{it}}$ and $r_{\mathrm{ref}}$ are adjustable parameters of the model, however during model validation/calibration these parameters are not the subject for optimization and we always use $\alpha_{\mathrm{it}}\;=\;10^{-13}\;$ cm ${}^{2}$ and $r_{\mathrm{ref}}\;=\;1\;$ nm.

### 2.4. Calibration of the Deterministic Model for HCD

**Figure 3:**
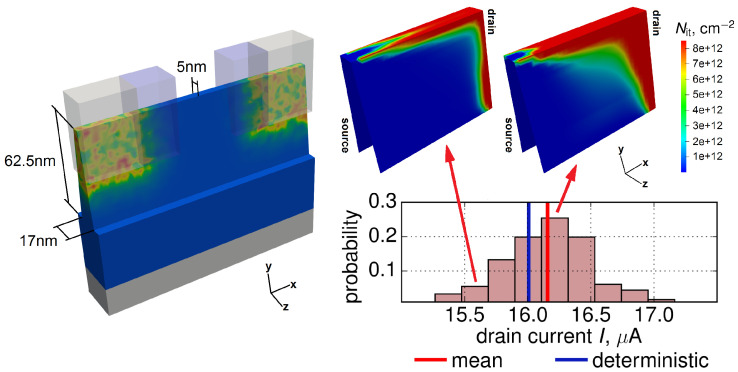
A sketch of the n-FinFET with random dopants (left), the distribution of the time-0 linear drain currents $I_{d,\mathrm{lin}}^{\left(0\right)}$ and $N_{\mathrm{it}}$ densities obtained at $V_{\mathrm{ds}}\;=\;$ 1.8 V, $V_{\mathrm{gs}}$ = 1.9 V and *t* = 200 s for two different $I_{d,\mathrm{lin}}^{\left(0\right)}$ values (right).

We employ n-channel FinFETs with a gate length of 40 nm (the corresponding channel length is 28 nm), a high-*k* gate stack (containing SiO${}_{2}$ and HfO${}_{2}$ layers) with an EOT of ∼1.2 nm, and an operating voltage of $V_{\mathrm{dd}}$ = 0.9 V. The FinFET geometry and dimensions such as the fin width and height are shown in Figure 3, left panel. To obtain the device architecture (including the doping profiles) we used the Sentaurus Process simulator [66], which was coupled to the device simulator MiniMOS-NT [63] in the GlobalTCADSolutions framework [67]. These two simulators were calibrated self-consistently by reproducing current-voltage characteristics of the pristine transistor; this is a very important step of the entire simulation framework because carrier energy distribution functions are very sensitive to variations in doping profiles.

**Figure 4:**
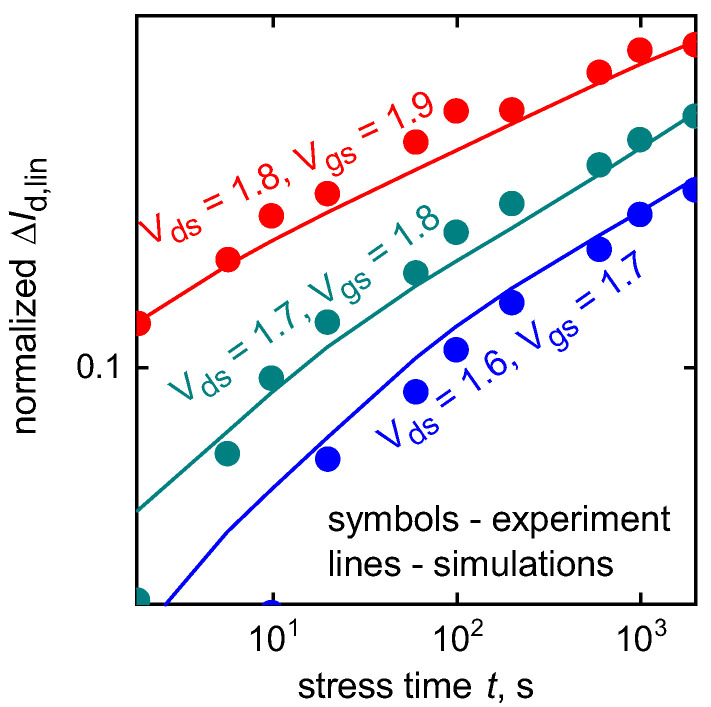
Experimental and simulation $\Delta I_{d,\mathrm{lin}}\left(t\right)$ degradation traces are in good agreement for all three stress conditions. The data are from [36].

These devices were stressed at the HCD worst-case conditions for short-channel devices for three different combinations of gate and drain voltages ($V_{\mathrm{gs}}$, $V_{\mathrm{ds}}$): $V_{\mathrm{ds}}\;=\;$ 1.6 V, $V_{\mathrm{gs}}$ = 1.7 V; $V_{\mathrm{ds}}\;=\;$ 1.7 V, $V_{\mathrm{gs}}$ = 1.8 V; and $V_{\mathrm{ds}}\;=\;$ 1.8 V, $V_{\mathrm{gs}}$ = 1.9 V. At each time step *t*, stress was interrupted to measure $\Delta I_{d,\mathrm{lin}}\left(t\right)$ changes ($I_{d,\mathrm{lin}}$ corresponds to $V_{\mathrm{ds}}$ = 0.05 V and $V_{\mathrm{gs}}$ = $V_{\mathrm{dd}}$); the corresponding experimental $\Delta I_{d,\mathrm{lin}}\left(t\right)$ traces are summarized in Figure 4. It is noteworthy that we consider relative changes of the drain current, i.e., absolute changes of linear and saturation currents were normalized to the current values of the unstressed samples $I_{d,\mathrm{lin}}^{\left(0\right)}$ and $I_{d,\mathrm{sat}}^{\left(0\right)}$ (which are different for various “samples”), respectively. Further, unless stated otherwise, under “normalized changes” in the current we understand $\Delta I_{d,\mathrm{lin}}\left(t\right)\;=\;\left|I_{d,\mathrm{lin}}\left(t\right)-I_{d,\mathrm{lin}}\left(0\right)\right|/I_{d,\mathrm{lin}}\left(0\right)$ and $\Delta I_{d,\mathrm{sat}}\left(t\right)\;=\;\left|I_{d,\mathrm{sat}}\left(t\right)-I_{d,\mathrm{sat}}\left(0\right)\right|/I_{d,\mathrm{sat}}\left(0\right)$. In some special cases we also employ normalization to the mean degradation values (e.g., $\Delta I_{d,\mathrm{lin}}\left(t\right)/\langle \Delta I_{d,\mathrm{lin}}\left(t\right)\rangle$). Let us also emphasize that typically drain currents decrease during HC stress, however, $\Delta I_{d,\mathrm{lin}}$ and $\Delta I_{d,\mathrm{sat}}$ values defined in the afore-described manner increase with time. Showing degradation chages in absolute values is commonly used for HCD characterization and modeling by many groups [56,68,69,70,71,72,73,74,75,76] because degradation traces presented in this form can be plotted on a log-log scale to enable empirical fitting. Figure 4 borrowed from our recent publication [36] demonstrates that our model can thoroughly represent experimental $\Delta I_{d,\mathrm{lin}}\left(t\right)$ traces for all three stress conditions.

Note finally that the model does not consider the impact of self-heating on HCD. Although we recently presented a simulation framework which models coupled hot-carrier degradation and self-heating in nanowire FETs [77], analysis of the impact of self-heating on HC induced variability is outside the scope of this paper.

### 2.5. The Stochastic Model for HCD

The device structure generated by Sentaurus Process with continuous doping profiles was used as a template to generate 200 instantiations with different configurations of discrete random dopants [23,25]. To achieve this goal, for each mesh cell of the initial device we multiplied the local doping concentration by the volume of this cell, thereby obtaining the mean value of the number of dopants contained in this cell. In each of these FinFET instantiations we consider the number of dopants in each cell as a stochastic variate and to generate 200 different realizations of this variate we use a Poisson random number generator with the mean value obtained from the device structure with continuous doping concentrations. This randomization procedure was applied to both donors and acceptors in all device segments including the source, drain, channel, source/drain extensions, etc.

Therefore, each of these 200 “samples” has its own unique configuration of RDs and, as a consequence, unique values of $I_{d,\mathrm{lin}}^{\left(0\right)}$ values, see Figure 3. For each of these instantiations we solved the Boltzmann transport equation, obtained carrier DFs, computed the corresponding interface state densities $N_{\mathrm{it}}$ (Figure 3) and generated ensembles of $\Delta I_{d,\mathrm{lin}}\left(t\right)$ and $\Delta I_{d,\mathrm{sat}}\left(t\right)$ traces (note that $I_{d,\mathrm{sat}}$ is measured at $V_{\mathrm{gs}}$ = $V_{\mathrm{ds}}$ = $V_{\mathrm{dd}}$). In addition to the aforementioned stress conditions, we carried out all these simulations for lower biases of $V_{\mathrm{gs}}\;=\;V_{\mathrm{ds}}$ = 1.0 V which are close to the operating voltage $V_{\mathrm{dd}}$.

The obtained continuous $N_{\mathrm{it}}$ densities were also a subject for discretization/randomization [24,26]. In a similar manner as in the case of RDs we multiplied the local $N_{\mathrm{it}}$ value by the cell area (a cell at the interface is two-dimensional though), thereby obtaining the mean number of traps which was then used in a Poisson randomizer to obtain the fluctuating number of interface traps in the cell. Special attention was paid that this randomization procedure does not result in a statistically scattered number of traps which is lower at the next stress time step as compared to the current step; in other words the discretized number of traps should be a non-reducing function of time.

To summarize, for each combination of stress voltages $V_{\mathrm{ds}}$, $V_{\mathrm{gs}}$ we generated 200 different configurations of RDs and then each of them, in turn, has 200 different realizations of RTs. Therefore our ensemble contained 40,000 different structures with unique RD and RT configurations.

Based on the initial 200 instantiations with RDs we calculated ensembles of $\Delta I_{d,\mathrm{lin}}\left(t\right)$ and $\Delta I_{d,\mathrm{sat}}\left(t\right)$ traces and extracted corresponding device lifetimes. For this extraction, we used the criterion that device lifetime corresponds to stress time at which the normalized $\Delta I_{d,\mathrm{lin}}$ (or $\Delta I_{d,\mathrm{sat}}$) reaches a value of 0.1. In the case of 40,000 different configurations of RDs and RTs we modeled only $\Delta I_{d,\mathrm{lin}}$ and corresponding lifetimes.

## 3. Results and Discussions

**Figure 5:**
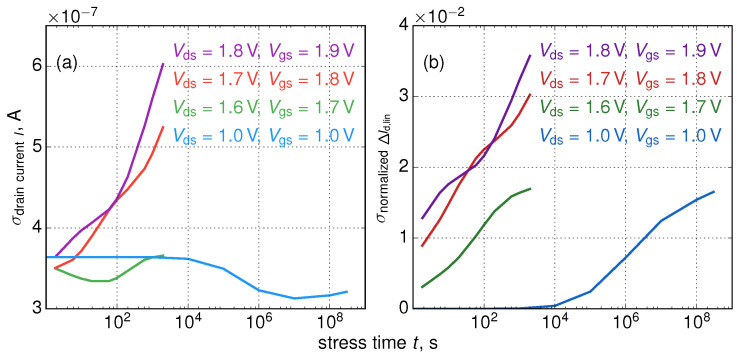
The standard deviations of the linear drain $I_{d,\mathrm{lin}}$ (**a**) and the relative linear drain current change $\Delta I_{d,\mathrm{lin}}$ (**b**) as functions of stress time *t*.

The distribution of the $I_{d,\mathrm{lin}}^{\left(0\right)}$ current is shown in Figure 3. As we discussed in [23,25,78], $I_{d,\mathrm{lin}}^{\left(0\right)}$ values are normally distributed and the current distribution broadens during HC stress. Such a trend can be seen in Figure 5a, which shows that the standard deviation of the linear drain current increases with stress time and this behavior is typical for all stress conditions. Quite interestingly, for much milder stress conditions with $V_{\mathrm{gs}}$ = $V_{\mathrm{ds}}$ = 1.0 V (close to the operating voltage) the standard deviation of $I_{d,\mathrm{lin}}$ decreases with stress time. This means that in the operating regime $I_{d,\mathrm{lin}}$ variability reduces and this peculiarity was reported in experimental papers by three different groups [29,31,79]. Note also that in our previous publications [23,25] we showed that degradation characteristics obtained for more aggressive HC stress conditions and at the milder operating regime obey distributions with different shapes. This is consistent with our current results.

As for the degradation traces $\Delta I_{d,\mathrm{lin}}\left(t\right)$, their ensemble also becomes broader as can be concluded from the standard deviation of $\Delta I_{d,\mathrm{lin}}$ which is a growing function of *t* (Figure 5b). Figure 3 depicts also interface state densities $N_{it}$ for $V_{\mathrm{ds}}\;=\;$ 1.8 V, $V_{\mathrm{gs}}$ = 1.9 V and *t* = 200 s evaluated for two devices, i.e., for one with $I_{d,\mathrm{lin}}^{\left(0\right)}$ equal to the mean value of the time-0 current and for a sample with a much lower initial current. One can see that in the former FinFET HCD is much more severe than in the latter device.

**Figure 6:**
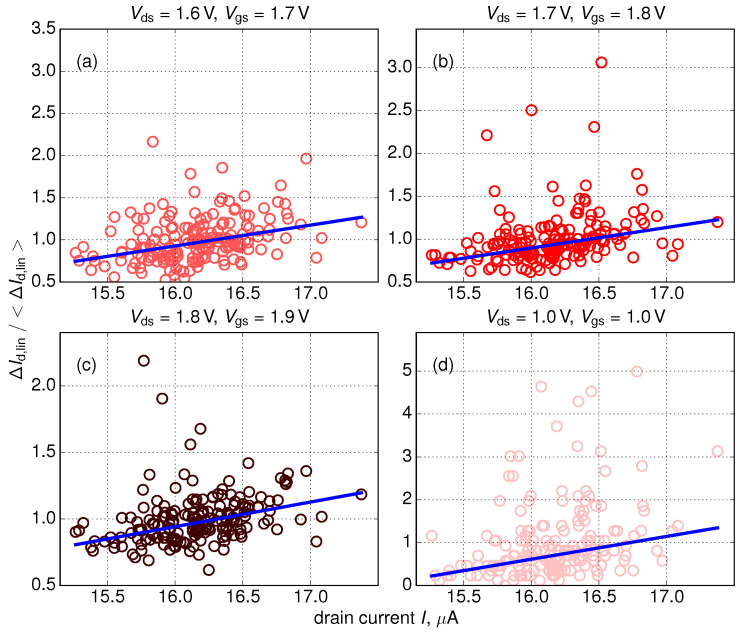
$\Delta I_{d,\mathrm{lin}}\left(t\right)/\langle \Delta I_{d,\mathrm{lin}}\rangle$ values plotted vs. $I_{d,\mathrm{lin}}^{\left(0\right)}$ currents. Data for all four stress conditions and stress time of 2 s are shown. Note that $\Delta I_{d,\mathrm{lin}}$ changes are normalized to the mean linear drain current change $\langle \Delta I_{d,\mathrm{lin}}\rangle$.

To study this tendency in greater detail for each of 200 instantiations we obtained $\Delta I_{d,\mathrm{lin}}$ changes and plotted them against $I_{d,\mathrm{lin}}^{\left(0\right)}$ current values, see Figure 6. Note that in Figure 6 we present additionally normalized changes $\Delta I_{d,\mathrm{lin}}\left(t\right)/\langle \Delta I_{d,\mathrm{lin}}\rangle$ (let us remind that $\Delta I_{d,\mathrm{lin}}$ itself is already normalized, i.e., $\Delta I_{d,\mathrm{lin}}\left(t\right)\;=\;\left|I_{d,\mathrm{lin}}\left(t\right)-I_{d,\mathrm{lin}}\left(0\right)\right|/I_{d,\mathrm{lin}}\left(0\right)$). Due to statistical distribution of RD positions these data are very scattered and therefore—in order to extract a possible correlation between $\Delta I_{d,\mathrm{lin}}\left(t\right)/\langle \Delta I_{d,\mathrm{lin}}\rangle$ and $I_{d,\mathrm{lin}}^{\left(0\right)}$—we utilized the robust linear fits employing the Kendall rank correlation coefficient [80]. Note that more advanced statistical techniques were avoided as those typically require preliminary information about the exact interrelation between variates.

**Figure 7:**
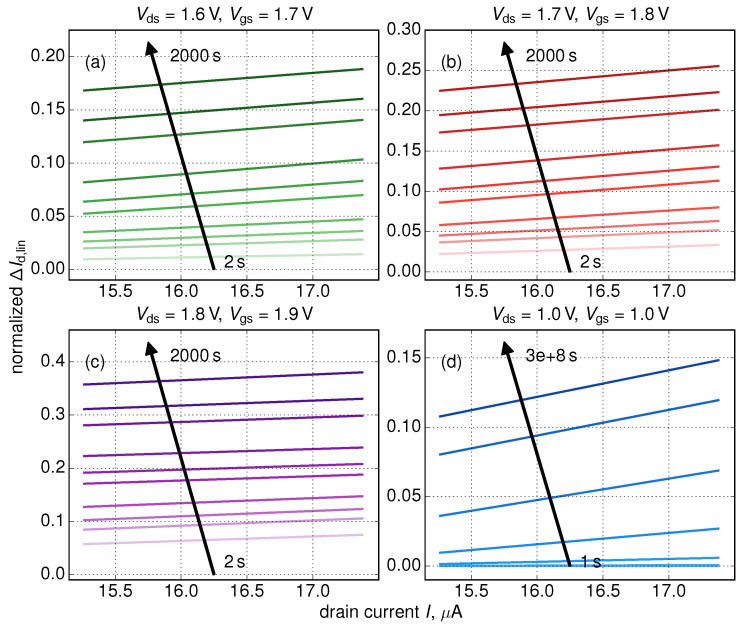
$\Delta I_{d,\mathrm{lin}}\left(I_{d,\mathrm{lin}}^{\left(0\right)}\right)$ dependencies for all stress time steps and four combinations of $V_{\mathrm{ds}}$, $V_{\mathrm{gs}}$. As opposed to Figure 6 showing additionally normalized $\Delta I_{d,\mathrm{lin}}\left(t\right)/\langle \Delta I_{d,\mathrm{lin}}\rangle$ traces this figure depicts relative changes of the linear drain current, i.e., $\Delta I_{d,\mathrm{lin}}\left(t\right)\;=\;\left|I_{d,\mathrm{lin}}\left(t\right)-I_{d,\mathrm{lin}}\left(0\right)\right|/I_{d,\mathrm{lin}}\left(0\right)$).

**Figure 8:**
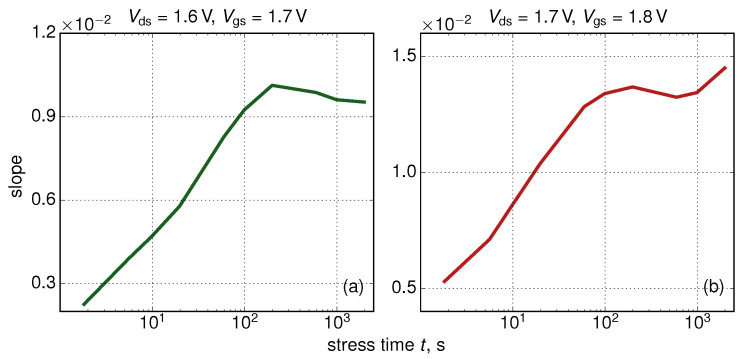
The slope of the $\Delta I_{d,\mathrm{lin}}\left(I_{d,\mathrm{lin}}^{\left(0\right)}\right)$ dependency as a function of stress time *t* for $V_{\mathrm{ds}}\;=\;$ 1.6 V, $V_{\mathrm{gs}}$ = 1.7 V and $V_{\mathrm{ds}}\;=\;$ 1.7 V, $V_{\mathrm{gs}}$ = 1.8 V.

This statistical analysis allowed us to extract the linear $\Delta I_{d,\mathrm{lin}}\left(t\right)/\langle \Delta I_{d,\mathrm{lin}}\rangle \left(I_{d,\mathrm{lin}}^{\left(0\right)}\right)$ dependency which shows that (on average) changes in the linear drain current have larger values in devices with larger $I_{d,\mathrm{lin}}^{\left(0\right)}$ currents. In other words, we conclude that initially more performing FinFETs degrade faster than their “worse” counterparts. This trend is typical for the entire time window, as can be seen from Figure 7, where $\Delta I_{d,\mathrm{lin}}\left(I_{d,\mathrm{lin}}^{\left(0\right)}\right)$ dependencies are plotted for all stress time steps. As for the slope of the $\Delta I_{d,\mathrm{lin}}\left(t\right)$ dependency (Figure 8), it increases at short stress times and then starts to decrease. This behavior is related to saturation of HCD at long times and/or high stress voltages [36,72]. Some further increase of this slope is visible in Figure 8b after ∼1 ks but we believe that this increase is related to a numerical artifact; and this increase is less than 10%.

**Figure 9:**
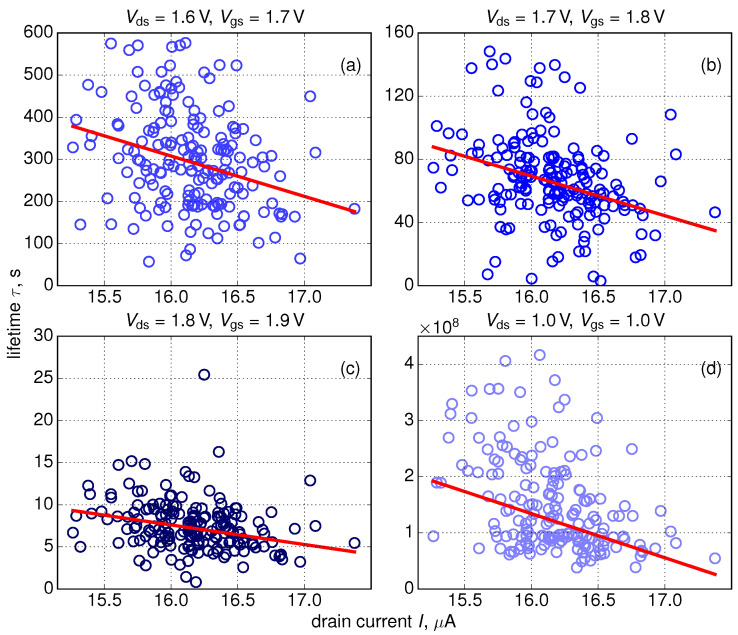
Extracted dependencies of device lifetime τ on $I_{d,\mathrm{lin}}^{\left(0\right)}$ for all stress conditions. To evaluate device lifetime values we used a $\Delta I_{d,\mathrm{lin}}$ = 0.1 criterion.

From the calculated $\Delta I_{d,\mathrm{lin}}\left(t\right)$ traces we extracted device lifetimes τ (τ is time at which $\Delta I_{d,\mathrm{lin}}$ = 0.1) and plotted them against currents in the pristine device $I_{d,\mathrm{lin}}^{\left(0\right)}$, see Figure 9. In the same manner as for the $\left\{\Delta I_{d,\mathrm{lin}},I_{d,\mathrm{lin}}^{\left(0\right)}\right\}$ tuples we employed the Kendall rank correlation coefficient and obtained the linear interrelation between τ and $I_{d,\mathrm{lin}}^{\left(0\right)}$ which is consistent with our findings shown in Figure 6 and Figure 7, i.e., FETs with higher $I_{d,\mathrm{lin}}^{\left(0\right)}$ values degrade faster and therefore have shorter lifetimes τ.

**Figure 10:**
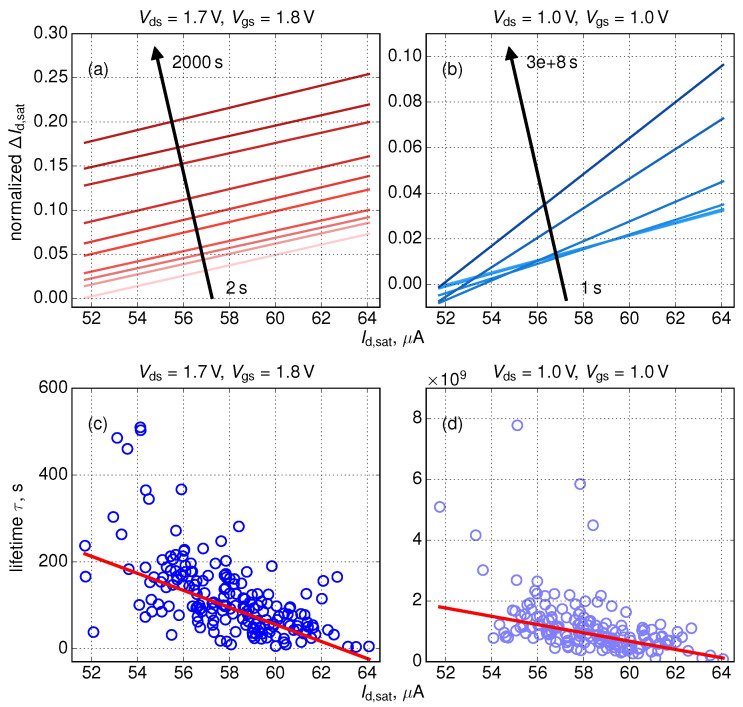
Extracted $\Delta I_{d,\mathrm{sat}}\left(I_{d,\mathrm{sat}}^{\left(0\right)}\right)$ dependencies for all stress time steps (**a**,**b**) and device lifetime obtained using $\Delta I_{d,\mathrm{sat}}$ traces (**c**,**d**).

All previous calculations were carried out based on $\Delta I_{d,\mathrm{lin}}\left(t\right)$ traces, but changes in the saturation drain current $\Delta I_{d,\mathrm{sat}}$ are another important metric of HCD. To show that all the trends obtained using $\Delta I_{d,\mathrm{lin}}\left(t\right)$ are typical also for $\Delta I_{d,\mathrm{sat}}\left(t\right)$ data we evaluated $\Delta I_{d,\mathrm{sat}}\left(I_{d,\mathrm{sat}}^{\left(0\right)}\right)$ dependencies for all stress times and two stress conditions with $V_{\mathrm{ds}}$ = 1.7 V, $V_{\mathrm{gs}}$ = 1.8 V and $V_{\mathrm{ds}}\;=\;V_{\mathrm{gs}}$ = 1.0 V, see Figure 10a,b. Using these data, lifetimes (based on the criterion of $\Delta I_{d,\mathrm{sat}}$ = 0.1) were extracted and summarized in Figure 10c,d. One can see that Figure 10 demonstrates exactly the same trends as those seen in Figure 7 and Figure 9. Let us note that typically $\Delta I_{d,\mathrm{lin}}$ values are larger than $\Delta I_{d,\mathrm{sat}}$ changes and therefore lifetimes extracted using the $\Delta I_{d,\mathrm{lin}}$ = 0.1 criterion are shorter than those which were obtained assuming $\Delta I_{d,\mathrm{sat}}$ = 0.1. As a consequence, the lifetime distributions presented in Figure 10c,d are shifted towards longer values as compared to the distributions shown in Figure 9b,d.

Stronger hot-carrier degradation featured by FinFETs with higher $I_{d,\mathrm{lin}}^{\left(0\right)}$ currents can be understood within our simulation framework considering coupled single- and multiple-carrier mechanisms of Si-H bond dissociation. The drain current density $j_{d}$ can be estimated as $j_{d}∼n\cdot V_{\mathrm{gr}}$, where *n* is the carrier concentration and $V_{\mathrm{gr}}$ the group velocity. A higher $j_{d}$ value demonstrated by a certain “sample” stems from a superposition of two factors: (i) carriers have either higher densities or (ii) higher velocities (i.e., they are hotter) as compared to another instantiation. Roughly, the former aspect results in an increased rate of the multiple-carrier mechanism, while the latter one can enhance both single- and multiple-carrier mechanisms of bond rupture. Therefore we conclude that both aspects lead to accelerated HCD.

**Figure 11:**
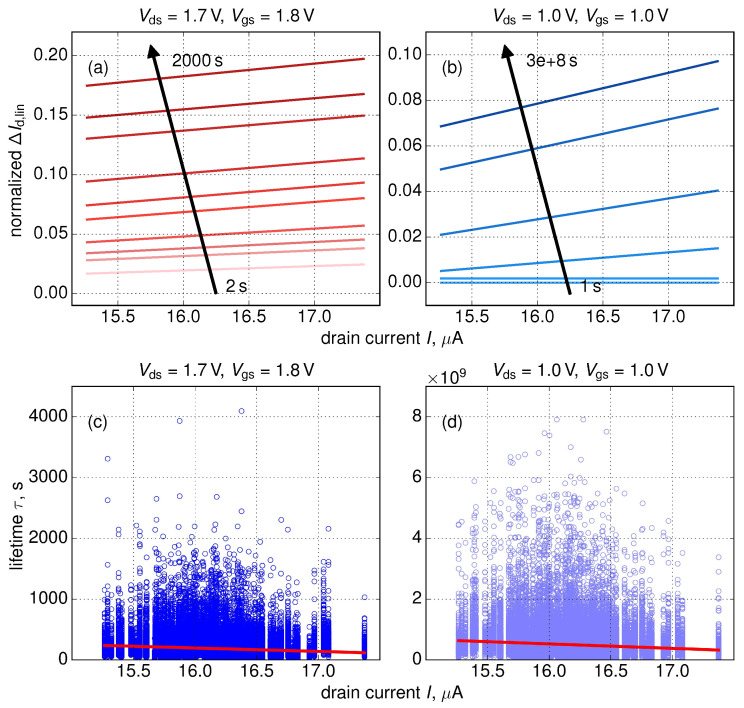
The same as in Figure 7a,b and Figure 9c,d but considering the impact of both RDs and RTs and for two stress regimes.

So far, we considered the impact of RDs on HCD, while the impact of random traps (RTs) was ignored. However, as we showed in our recent publications [24,26], RTs result in different shapes of $\Delta I_{d,\mathrm{lin}}$ and τ distributions. Nevertheless, Figure 11 shows that $\Delta I_{d,\mathrm{lin}}\left(I_{d,\mathrm{lin}}^{\left(0\right)}\right)$ and $\tau (I_{d,\mathrm{lin}}^{\left(0\right)})$ dependencies obtained considering impacts of both RDs and RTs show the same trends as those curves generated taking only RDs into account. Note that the impact of RTs results in broadening of τ distributions, see [24,26], but this trend is a natural consequence to the fact that inclusion of RTs into our modeling approach adds an additional source of variability.

## 4. Conclusions

Using our statistical model for hot-carrier degradation, we generated an ensemble of 200 instantiations of the n-channel FinFET with unique random dopants configurations, calculated time-0 drain currents, their normalized changes with stress time, and extracted device lifetimes. Then, current changes were plotted vs. time-0 currents and the robust linear fit employing the Kendall rank correlation coefficient allowed us to identify correlation between these two variates. Our correlation analysis has shown that FinFETs with higher time-0 currents degrade faster and therefore have larger current changes and shorter device lifetimes. This qualitative behavior holds true for both linear and saturation drain currents (and corresponding lifetimes) and is consistent with the similar trend previously reported for bias temperature instability. Furthermore, we have shown that the impact of random traps does not changes all the aforementioned tendencies. Finally, we have showed that under stress conditions with high voltages the current distribution spreads with stress time, while in the much milder operating regime the variability of the drain current decreases. This trend agrees with our previous results showing that degradation characteristics have different distributions in stress and operating regimes.

## Abbreviations

The following abbreviations are used in this manuscript:

BTE	Boltzmann transport equation
DFs	distribution functions
FET	field-effect transistor
HCD	hot-carrier degradation
MC	multiple-carrier (machanism of bond rupture)
RDs	random dopants
RTs	random traps
SC	single-carrier (mechanism of bond rupture)
